# Out of control: computational dynamic control dysfunction in stress- and anxiety-related disorders

**DOI:** 10.1007/s44192-023-00058-x

**Published:** 2024-01-18

**Authors:** Jonathon R. Howlett, Martin P. Paulus

**Affiliations:** 1 VA San Diego Healthcare System, 3350 La Jolla Village Dr, San Diego, CA 92161 USA; 2 Department of Psychiatry, University of California San Diego, La Jolla, CA USA; 3 Laureate Institute for Brain Research, Tulsa, OK USA

## Abstract

Control theory, which has played a central role in technological progress over the last 150 years, has also yielded critical insights into biology and neuroscience. Recently, there has been a surging interest in integrating control theory with computational psychiatry. Here, we review the state of the field of using control theory approaches in computational psychiatry and show that recent research has mapped a neural control circuit consisting of frontal cortex, parietal cortex, and the cerebellum. This basic feedback control circuit is modulated by estimates of reward and cost via the basal ganglia as well as by arousal states coordinated by the insula, dorsal anterior cingulate cortex, amygdala, and locus coeruleus. One major approach within the broader field of control theory, known as proportion-integral-derivative (PID) control, has shown promise as a model of human behavior which enables precise and reliable estimates of underlying control parameters at the individual level. These control parameters correlate with self-reported fear and with both structural and functional variation in affect-related brain regions. This suggests that dysfunctional engagement of stress and arousal systems may suboptimally modulate parameters of domain-general goal-directed control algorithms, impairing performance in complex tasks involving movement, cognition, and affect. Future directions include clarifying the causal role of control deficits in stress- and anxiety-related disorders and developing clinically useful tools based on insights from control theory.

## Introduction

Parsing how biological, psychological, and social mechanisms contribute to stress- and anxiety-related disorders is essential for the identification of specific etiological factors and pathways. This precise knowledge is critical in not only formulating but also tailoring novel interventions, which could range from pharmacological strategies, leveraging advances in neuropharmacology, to cognitive-behavioral therapies, which address maladaptive thought patterns and behaviors [1]. The emerging field of computational psychiatry facilitates this parsing process by developing explicit, generative models of the internal processes that generate observable behaviors, enabling insight into individual processing differences that may drive psychiatric disorders [2]. Much of the effort in computational psychiatry has been focused on reinforcement learning models of decision-making tasks in which a decision is typically made once every several seconds [3]. This research program has led to improved understanding of the mechanisms of a range of psychiatric disorders, including those related to stress and anxiety [4–10]. However, there has been increasing recognition of the need to improve reliability and construct validity of individual measures derived from computational models for these models to be clinically useful [11].

Emerging research suggests that computational psychiatry may benefit from incorporating insights from the field of control theory. Control theory is a branch of mathematics and engineering that deals with the behavior of dynamic systems. The main objective of control theory is to design systems in a way that they can operate in a desired manner. In simple terms, a dynamic system is any system that changes over time, which could be as simple as a thermostat regulating room temperature, or a complex system like a self-driving car navigating a city. Control theory focuses on the rules (or “controllers”) that guide the system's behavior to reach a particular goal. This often involves maintaining a certain state (like keeping a room at a certain temperature) or following a desired path (like a self-driving car reaching its destination). Control theory uses feedback, which is information about the current state of the system, to adjust the input to the system to achieve the desired output. For example, if a room is too cold, the thermostat gets this feedback, and increases the heat input to reach the desired temperature. While the concept is simple, the actual practice of control theory can be quite complex, involving advanced mathematics to handle systems with multiple inputs and outputs, non-linear behaviors, and uncertainties. Control theory has influenced engineering, economics, computer science, and many other disciplines.

There is a deep relationship between the mathematical foundations of control theory and reinforcement learning [12], and recent analyses suggest that control theoretic algorithms may represent a class of domain-general neural mechanisms involved in an array of cognitive processes. For example, the Bellman equation in reinforcement learning has a counterpart in the Hamilton–Jacobi–Bellman equation in control theory [13]. Both equations provide a recursive framework for optimal decision-making. This mathematical similarity suggests that algorithms developed in control theory could be applied to RL problems and vice versa. Moreover, recent research has begun to explore how control theoretic algorithms may represent a class of domain-general neural mechanisms [14]. For example, control theory specifies a domain-general process in which state estimates are updated in response to new information and then used to drive adaptive actions in response to the current state. While sensorimotor control relies on concrete state variables such as physical position and velocity, abstract planning and cognitive control may involve abstract latent state variables tracking progress toward abstract goals. Similar computational neural processes may govern the updating of state estimates and the use of these estimates to drive actions for both concrete and abstract state variables. Control theory algorithms have recently been applied to probabilistic learning and decision-making [14] and could lead to a more unified account of neural computational processing across domains. In the context of mental health, improved models of these domain-general neural mechanisms could be crucial for understanding conditions like anxiety, depression, and substance use disorders. Maladaptive decision-making is a common feature in these conditions, and understanding the neural basis of decision-making through the lens of control theory and reinforcement learning could offer novel treatment targets or prognostic biomarkers. It could also contribute to the development of transdiagnostic interventions that improve patient outcomes by targeting these domain-general neural mechanisms. Moreover, the computational motor control literature has demonstrated that sophisticated computational algorithms underpin the control of movement in humans [15], leading to the recognition that real-time sensorimotor control should be understood as a complex form of statistical decision-making in the same vein as traditional reinforcement learning tasks [16]. These developments are particularly relevant to individual difference research in computational psychiatry given that real-time, continuous behavioral data from sensorimotor tasks (e.g. those using joysticks or touchscreens) can consist of hundreds of data points gathered over several seconds rather than a single decision data point, potentially yielding far more reliable parameter estimates in a much shorter time [17].

Control theory has been a major conceptual influence in biology since the early twentieth century [18] via concepts such as homeostasis (maintenance of favorable conditions through feedback control) and allostasis (anticipatory adaptations supported by stress and arousal responses). Allostatic processes, mediated by stress hormones and catecholamines such as norepinephrine (NE), are activated in response to stressors or disturbances which overwhelm normal homeostatic mechanisms [19]. Dysfunction related to arousal and stress may be particularly well understood through the lens of allostasis as a dysfunction of adaptive control mechanisms affecting movement [20] and cognitive processes [21]. Control theory could therefore represent a valuable tool for understanding dysfunction of arousal and stress systems and resulting impaired processing in stress- and anxiety-related disorders. In this review, we will introduce a computational control theory framework for understanding mechanistic dysfunction underlying stress- and anxiety-related disorders. We will begin with a brief and highly selective introduction to some relevant topics in control theory, including proportional-integral-derivative (PID) control, adaptive control, and state estimation. Note that a comprehensive overview of control theory is outside the scope of this paper; interested readers are referred to relevant textbooks [22, 23]. Next, the role of control theory in biology will be introduced, focusing on homeostasis and computational models of motor control. The influence of motivation on movement will be reviewed, along with the role of arousal in modulating parameters of control and state estimation. Control circuits will be examined as implementing domain-general goal-directed control algorithms which support regulation of cognition and affect as well as movement. We will examine recent empirical evidence for associations between individual differences in control parameters and self-reported fearful affect as well as neural structure and function. We will conclude by proposing a framework for understanding dysfunctional control processing as both consequences and causes of stress- and anxiety-related disorders. Given the early stage of this research, we will not examine disorders as separate diagnostic categories but rather use a dimensional transdiagnostic approach to a control deficit in anxiety consistent with the Research Domain Criteria (RDoC) proposed by the National Institute of Mental Health [24].

Historically, control theory has developed through several stages, including pre-classical and classical control theory in the early twentieth century and optimal control theory in the latter twentieth century [25]. Optimal control consists of finding control actions that maximize a performance objective based on an explicit model of a system [23], while earlier techniques such as PID control are more heuristic and do not require an explicit model [26]; both techniques are currently in wide use in industry. While this review will focus on PID control rather than optimal control, it is likely that these will be complementary approaches in understanding neural processes relevant to psychiatric disorders. By analogy, computational psychiatry makes use of both optimal algorithms (such as ideal Bayesian observer models) as well as heuristic algorithms (such as model-free reinforcement learning algorithms) [27].

## Control theory

### Proportional control

Control systems have been central to technological progress since the Industrial Revolution and play central roles in manufacturing, process automation, transportation, aerospace, and other industries [25]. These systems typically use *feedback control*, which allows for continuous monitoring and adjustment of system variables to achieve desired performance or outcomes [28]. Feedback control systems often function by comparing the current state of the environment (based on input from *sensors*) to a desired *reference value* and thereby computing a *reference error* that is used to adjust actions.

Proportional control is one of the simplest and oldest feedback control techniques [25]. In proportional control, a control signal is applied to reduce the difference between a current measured process variable and the desired reference value. The control signal equals the difference between the reference and the current value (i.e. the reference error), multiplied by a constant scaling or *gain* factor. As the control signal is applied, the difference between the actual and desired value is reduced, until the system is in the desired state and the control signal is reduced to zero.

### Proportional-integrative-derivative (PID) control

Despite the usefulness of proportional control, it has important limitations [26]. First, overshoot and oscillations can occur if the gain factor is too high. Overshoot occurs when the output of the system exceeds the desired setpoint before settling down to the steady-state value, similar to a pendulum swinging through its equilibrium position. If the gain factor is set too high, the system can be overly responsive and oscillate around the setpoint via a series of overcorrections (sometimes known as *hunting* behavior in control systems). To avoid oscillations, the proportional gain must be set to a lower value, thus slowing error correction and reducing the performance of the controller.

A second limitation of proportional control is the inability to eliminate steady state error in the presence of a constant disturbing force [26]. Steady-state error is a measure of the residual error between the setpoint and the measured process variable once the system has reached a stable operating point. For example, a proportional steering system for a ship would adjust the rudder to correct for the error, i.e. the difference between the actual direction or heading and the desired direction (reference heading). As the heading changes, the rudder is continually adjusted in proportion to the current error until the actual heading matches the reference heading. However, if there is a constant disturbance (such as a steady current) continually pushing the ship off course, proportional control can fail to reduce the error to zero and instead result in a nonzero steady-state error.

These limitations are addressed by proportional-integral-derivative (PID) control, which was developed in 1922 [29] and is now among the most widely used techniques in industry in fields such as manufacturing, chemical reactors, robotics, automation, and process control [26]. First developed by analyzing the performance of human pilots of naval ships, PID control adds two additional terms to augment the proportional control mechanism. First, derivative control adds a term that is proportional to the rate of change of the reference error signal. This term helps stabilize the system by anticipating and counteracting overshoot. Derivative control is analogous to friction in a physical system (such as a pendulum), adding a damping action that reduces overshoot and oscillations for a given level of proportional gain. This enables the controller to achieve higher proportional gain and faster performance while preventing overshoot and oscillations. Second, integral control adds a term that is proportional to the accumulated reference error signal over time. This term helps to eliminate steady-state errors that can occur due to system biases or external disturbances. PID control involves three gain terms known as *K*_*p*_, *K*_*i*_, and *K*_*d*_, i.e. the coefficients that are multiplied by current error, integral of the error, and derivative of the error, respectively. Generally, these open parameters are set by a process known as tuning, to achieve the best possible performance for a given system [30].

### Adaptive control

Generally, the optimal parameters in control systems (such as PID gain parameters) depend on the specific environment rather than having fixed global values. To address this, the field of adaptive control techniques allow control systems to continuously modify control parameters and strategies based on previous information, current conditions, and anticipated future changes, thus enabling efficient responses to dynamic changes in an environment [31]. For example, the gain parameters of PID controllers can be adaptively adjusted in response to environmental disturbances, or adjusted predictively before an expected environmental change (a process known as *gain scheduling*) [26]. Recent approaches to adaptive control include reinforcement learning, Bayesian inference, and machine learning techniques such as deep neural networks [31].

### State estimation

In order to determine appropriate control actions, feedback control systems require information about the current state of the environment (which they may then compare to a reference value to generate a control signal). However, in real systems, sensor signals are noisy and are time-lagged by the time they reach the controller. In order to address the problem of imperfect measurement, control systems can use *state estimation* techniques to combine incoming sensor values with other information to improve the accuracy of state information used to generate control signals [32]. For example, noisy sensor signals can be integrated with model-based predictions of the current state based on past measurements to yield a more accurate estimate. These techniques are often classified as *filters* as they remove noise from a sensor signal. A seminal example is the *Kalman filter*, which is the optimal state estimation technique in the setting of a linear system model and Gaussian noise [32]. In the Kalman filter, a model prediction of the current state is generated by applying a forward transition model (a set of linear differential equations) to the previous state estimate. Global Positioning System (GPS) is a practical example of the use of a Kalman filter. GPS receivers get signals from multiple satellites, each providing an estimate of the distance between the receiver and the satellite. Since the satellites’ locations are known, this distance data should allow the receiver to calculate its own position. However, these distance measurements are not perfect, due to factors like atmospheric noise, clock errors, and signal reflection. The Kalman filter is an algorithm that combines the noisy measurements from the satellites with a predictive model of the receiver’s motion, to estimate the most likely current position and velocity of the receiver. As new measurements come in, the Kalman filter updates its estimates in a way that minimizes the estimated error, given the statistical properties of the measurement noise and the uncertainties in the motion model. This results in a more accurate and reliable estimate of the receiver's location and velocity than would be possible using the measurements alone. The degree of reliance on the model prediction vs. the current measurement is determined by the *Kalman gain*, which depends both on the uncertainty of the model prediction and the uncertainty of the current measurement. When Kalman gain is low, the new estimate is based largely on the model prediction, while when Kalman gain is high, the new estimate is based largely on the new measurement. Kalman gain tends to be higher in a volatile environment, i.e. one which changes unpredictably, and tends to be lower in the setting of high sensor noise.

### Relationship to other concepts

The problem addressed by control theory (guiding a system’s behavior to reach a goal or maximize an objective function) potentially encompasses a broad array of neural functions. Control theory is therefore closely related to a number of other mathematical frameworks for studying neural computation, which, like control theory, are also generally rooted in mathematical optimization. As discussed above, the control problem can be separated into state estimation and the control law, with the Kalman filter being the optimal Bayesian inference technique for state estimation for a linear dynamical system with Gaussian noise [32]. The Kalman filter is one of a larger set of Bayesian techniques which have been used to model inference in neural systems. Specifically, the Kalman filter has been widely used to model associative learning, including associative reward learning [33]. In fact, the Kalman filter can be understood as an extension of the classic Rescorla-Wagner (RW) learning rule (which implements error-driven updates scaled by a learning rate) [34]. The RW learning rule is identical to the Kalman filter state update equation for a one-dimensional system without dynamics, except that the Kalman gain has been replaced by the fixed, heuristically determined learning rate rather than being optimally determined according to uncertainty in predictions and measurements. The Kalman filter is also closely related to active inference and free energy minimization, a general paradigm for inference and control in neural computation [35]. Free energy minimization can be viewed as a generalization of Bayesian filtering, of which the Kalman filter is a special case [36]. Finally, the Kalman filter is a particular case of model-based inference. Model-based inference involves predictions based on a model specifying how the environment transitions from one state to another. In the particular case of the Kalman filter, this model is represented by a set of linear differential equations. More generally, the extended Kalman filter, unscented Kalman filter, and particle filter can be applied to nonlinear state transitions [37]. When the model is not known a priori, model-based reinforcement learning techniques can be used to learn a model from experience (model-based reinforcement learning can also be used to learn an approximately optimal control law when a model is known) [38].

In addition to state estimation, another major component of a control system is the control law, which considers both the state estimate and the reference value (or objective function) to generate a control action. This aspect of the control problem is also closely related to other frameworks, including reinforcement learning and active inference, in which actions are selected to maximize an objective function. As in reinforcement learning, the Bellman equation (or its continuous version, the Hamilton–Jacobi–Bellman equation) represents the optimal policy for general control problems, but these equations are computationally costly to solve, necessitating simplifications in complex environments [13]. Interestingly, in a linear-quadratic-Gaussian (LQG) setting (i.e. a system with linear dynamics, a quadratic objective function, and Gaussian noise), there is a duality in the estimation and control problem, such that finding the optimal control law is equivalent to state estimation using a Kalman filter [39]. This analogy between estimation and control is also relevant to the active inference paradigm, in which action selection is treated formally as a probabilistic inference problem [36]. More heuristically, it can be noted that proportional control (in which the reference error is multiplied by a scaling term to generate the control action) resembles the RW learning rule (in which the prediction error is multiplied by a scaling term to generate the belief update). This analogy has been further extended by applying PID control to learning problems [14]. Conceptually, this distinction between prediction errors (in which the current state is compared to an expectation) and reference errors (in which the current state is compared to a goal) may be highly relevant to understanding neural computation.

## Control theory in biology: homeostasis and the control of behavior

### Control theory and homeostasis

In parallel to its application to technological systems, control theory has played a pivotal conceptual role in modern biology [18]. The parallel nature of control mechanisms in living organisms and machines was codified in the field of *cybernetics*, named after the Greek word for the steersperson of a ship [40], which has contributed to the study of a variety of biological processes such as gene networks, metabolism, blood composition, body temperature, and hormone secretion, as well as the neural control of behavior [41]. Control theory contributed directly to the concepts of *homeostasis* [42], the favorable range of internal and external conditions maintained through feedback control mechanisms, and *allostasis* [43], involving maintenance of stability through anticipatory adjustments for upcoming challenges via activation of stress response systems such noradrenergic arousal pathways. Among the biological systems subserving the goal of maintaining homeostasis, the voluntary motor system plays a central role, regulating physiological processes via behaviors such as feeding, drinking, and thermoregulation. Therefore, the control of movement has been a major focus for the application of control theory to biology.

### Computational motor control

Motor control is a natural application of control theory to biology as it involves goal-oriented activity which makes use of sensory feedback [44]. There is a long history of application of control theory to motor control beginning with cybernetics [40]. More recently, a sophisticated optimal control framework has been developed and applied empirically to study motor control in humans and animals [15, 16, 45, 46]. This approach emphasizes both similarities and differences between engineered (e.g. robotic) and biological systems, addressing special computational problems such as lagged, noisy feedback and the redundancy of motor systems (i.e. many possible movements to reach the same goal) in organisms. The control theory framework has been supported by empirical evidence such as variance in reaching movements toward a goal, effects of perturbation on movement, and the effect of manipulating rewards and costs of movements [15].

Recent research has integrated computational accounts of state estimation and control with neuroanatomy and physiology to infer a hypothesized neural circuit governing movement [47]. In this view, two central controllers incorporate sensory feedback to adjust motor commands. Primary motor cortex, which receives proprioceptive information from primary somatosensory cortex and the thalamus, is the feedback controller for the proprioceptive modality. Premotor cortex, which receives visual signals from posterior parietal cortex, is the feedback controller for the visual modality. Given the time lag for sensory signals to reach the brain, these controllers do not act on sensory input directly but instead receive current state estimates from parietal cortex. In turn, state estimation is performed in parietal cortex by combining incoming sensory information with predictions generated by an internal *forward model* implemented in the cerebellum [48]. Motor commands generated by motor and premotor cortex are projected to the spinal cord to produce movements and are also relayed to the cerebellum as inputs for the forward model for ongoing state estimation.

### Motivational influences on motor control

Given that the voluntary movement system evolved to serve higher-level goals related to preserving homeostasis or otherwise improving biological fitness, it is unsurprising that motor control systems are influenced by motivational systems in the brain. Evidence has shown that the expected rewards and costs associated with a movement are directly incorporated into the motor control loop itself. Basal ganglia may use the estimated value of an action to directly adjust the gains of sensorimotor feedback loops to optimize performance [47]. Supporting this, both animal and human movements are dependent on the potential rewards and costs of possible outcomes, and modulation of the basal ganglia (via anatomic lesions, Parkinson’s Disease, or pharmacologic agents affecting the dopamine system) alter movement vigor, potentially by biasing estimates of the rewards vs. costs associated with action [49, 50].

### Influence of stress and arousal on motor control

In response to and anticipation of challenges to homeostasis, organisms mount allostatic responses which alter the functioning of organ systems throughout the body. A set of neural regions termed the salience network, comprising the amygdala, insula, and dorsal anterior cingulate cortex (dACC), are critical for perceiving and responding to homeostatic demands, in part by coordinating the arousal response [51]. NE is a central mediator of this global arousal response and is released throughout the central nervous system via projections from the locus coeruleus (LC), with particularly dense projections to sensorimotor control regions [52]. The presence of these extensive modulatory pathways suggests that allostatic arousal is an evolutionarily conserved form of adaptive control, altering sensorimotor control laws in response to salient internal or external signals.

A unifying account of the diverse effects of NE across regions and cortical layers is the adaptive gain theory, which posits that NE sharpens the gain of neural signals by enhancing the activity of strongly activated neurons while suppressing the activity of weakly activated neurons, thus amplifying and speeding the response to incoming signals [53]. Computational analyses have supplemented this view by suggesting that NE conveys an “unexpected uncertainty” signal, conveying an increased expectation of environmental volatility and leading to stronger reliance on new information rather than relying on internal models or averaging over past events [54–56]. In state estimation terms, this suggests that increased NE could signal increased state volatility, leading to an increased Kalman gain (or related parameter) which speeds responses to true changes at the expense of amplifying measurement noise.

Empirical research has confirmed that stress and arousal have extensive effects on movement and motor control. Much of the research is consistent with a proposed “inverted U” relationship between arousal and performance, initially demonstrated for perceptual discrimination [57] and later extended to other domains including motor control. The optimal arousal level varies for different motor tasks, with high arousal levels being optimal for tasks involving strength, endurance, and speed, and moderate arousal levels being optimal for tasks involving complex skills, fine muscle movements, coordination, and steadiness [20]. Experimental manipulations increasing arousal or anxiety have been shown to disrupt performance in a range of complex tasks including wall climbing [58], putting in golf [59], and target shooting [60]. Taken together, the evidence for an inverted U relationship between arousal and motor control performance, together with the observation that the optimal level of arousal depends on the task and context, is consistent with an account in which the arousal system implements a form of adaptive control which modulates control parameters (such as Kalman gain) to match the situation (with high arousal being beneficial for simple tasks and moderate arousal being beneficial for complex tasks).

### Parallel neural circuits for goal-directed control

A striking feature of cerebral organization is the presence of a series of parallel circuits subserving different functions. These circuits include parallel frontostriatal loops, in which cortex projects to the basal ganglia (through the striatum, their input nucleus), which projects to the thalamus, which projects back to cortex [61]. The parallel loops include motor and oculomotor circuits as well as a prefrontal circuit and a limbic circuit. The functions of these largely segregated frontostriatal loops range from motor control and planning, working memory, cognitive control, and executive function, to reward processing and emotion regulation. Supporting this, damage to or dysfunction of frontostriatal circuits causes not only motor deficits but also executive dysfunction, behavioral disinhibition and emotional lability, and motivational impairment [62]. Given the strong resemblance in organization within each segregated loop, the fundamental processing operations within each circuit are believed to be similar [61].

In addition to the basal ganglia, the frontal cortex is also connected to the cerebellum in a similarly constructed loop. Along with input from sensorimotor cortical systems, the cerebellum also receives input from dorsolateral and medial prefrontal cortices, frontal language regions, posterior parietal cortices, superior temporal cortex, anterior cingulate cortex, and posterior hypothalamus [63]. The cerebellum projects back to these same cortical areas via the thalamus. Cerebellar damage can cause a syndrome comprised of cognitive and affective changes including executive dysfunction, deficits in spatial cognition and language, blunted affect, and behavioral disinhibition [64]. As in the frontostriatal loops, the cerebellum consists of a repeated canonical circuit, with one pathway consisting of mossy fibers projecting to Purkinje cells, which in turn project to dentate cells, along with a separate pathway in which climbing fibers project to Purkinje cells. This circuit likely implements similar computational functions across a range of domains, although recent evidence has highlighted diversity within these circuits that may enable functional differences [65].

The parallel organization of circuits involved in motor control, motor planning, working memory, cognitive control, and emotion regulation suggest that similar computational algorithms underlie these various cerebral functions. More specifically, computational algorithms which originally evolved for balance and movement may have subsequently been co-opted for more abstract cognitive functions. These considerations raise three intriguing possibilities. First, model-based state estimation in cerebellum and parietal cortex: the cerebellum and parietal cortex are involved in tasks related to motor control and spatial awareness. The use of model-based state estimation, like the Kalman filter, suggests that these regions continually estimate the current state of the body (or cognitive process) based on incoming sensory data and previous states. This is akin to predicting the current position and velocity of a physical system based on previous measurements and a model of the system’s dynamics. Second, feedback control in frontal cortex: the frontal cortex may adjust behavior or cognitive processes based on the difference between the intended goal and the current state. This is similar to how a thermostat uses feedback to maintain a desired temperature. Third, modulation by reward and cost in the basal ganglia: the basal ganglia are involved in various functions, including motor control, learning, and reward-based decision making. In the context of control theory, this suggests that the basal ganglia play a role similar to a cost function optimizer, adjusting the inputs to the system (i.e., decisions or actions) based on the perceived rewards and costs of different options.

While currently speculative, the hypothesis that cognitive control and emotion regulation involve state estimation based on a forward model combined with feedback control to regulate responses is based on multiple lines of evidence. The idea that the cerebellum acts as a “future predictor” in the brain has been around for a long time and is supported by various lines of research, including studies on brain structure, animal and human behavior, brain scans, clinical studies, and non-invasive stimulation [66–68]. Activity in cerebellar output cells (dentate cells) linearly predict future activity in cerebellar input cells (mossy fibers), consistent with a Kalman filter-like computation in which Purkinje cells (which receive inputs from mossy fibers and provide outputs to dentate cells) compute state predictions based on mossy fiber inputs [69]. While the nature of cerebellar computation is clearest for motor processing, the parallel nature of the mossy fiber-Purkinje cell-dentate cell circuit suggests that a similar computation underlies the role of the cerebellum in cognitive and affective processing [68]. The observation that frontal brain areas responsible for cognitive control and emotion regulation receive signals from the cerebellum implies that these frontal regions use real-time information from the cerebellum to perform calculations. Specifically, it suggests that these frontal areas are engaged in a form of feedback control, integrating current state estimates into their computational processes. The specific nature of these computations is poorly understood, and future research is needed to clarify the role of feedback control in prefrontal functioning (similar to modeling work examining parallel striatal computations involved in action selection, working memory, and executive function [70, 71]). These investigations can build on recent studies applying control theory algorithms to non-motor cognitive processing [14].

It is important to note that hypotheses regarding the role of domain-general control theory algorithms in abstract cognitive processes remain speculative and unproven. In the emerging intersection of control theory and computational psychiatry, a comprehensive research program will need to be developed to systematically examine the role of domain-general control theory in psychiatry. At the core of this program is the development of a detailed computational framework, informed by PID control theory, to model the interactions among the frontal cortex, parietal cortex, and cerebellum during cognitive tasks. This framework will need to be designed to incorporate the modulatory influences of stress and arousal systems and to examine the role of the insula, dorsal anterior cingulate cortex, amygdala, and locus coeruleus on control parameters. Through a blend of simulation studies in virtual environments and empirical cross-domain comparisons, this program will need to dissect how these control mechanisms vary under different states of stress and arousal and how they correlate with individual differences in fear and anxiety. The integration of behavioral data with functional magnetic resonance imaging (fMRI) through machine learning techniques will need to be used to validate and refine the PID control models. Moreover, transdiagnostic studies will need to be conducted to probe the variation of control parameters across psychiatric conditions, seeking biomarkers for clinical interventions. Longitudinal and interventional studies will need to be conducted to assess the temporal evolution of control parameters, providing predictive insights for treatment responses. This structured program not only promises to elucidate the computational underpinnings of psychiatric disorders but also to pave the way for novel diagnostic and therapeutic strategies.

## Control theory and individual affective differences

### Potential for a continuous control theory paradigm in computational psychiatry

A major function of the nervous system is to apply limited resources to rapidly interpret ambiguous sensory information and select and execute appropriate actions to meet the goals of the organism. Computational psychiatry aims to precisely characterize dysfunction in this process in individuals with mental health difficulties. As discussed above, control theory is one of several related frameworks to approach this general problem. Computational psychiatry studies often use binary-choice paradigms (such as two-armed bandits, change point detection tasks, and two-stage tasks) or go/no-go paradigms. While these paradigms have yielded important insights into reward processing, statistical inference, model-based vs. model-free decision making, and inhibitory control, they also exhibit important limitations.

First, using a control theory framework helps to emphasize the challenges involved in choosing actions within expansive or continuous sets of possible actions, a situation commonly encountered in real-world problems. This focuses attention on the control law, which selects from a range of possible control actions based on the state estimate. By contrast, in binary-choice paradigms, the focus is on the process of estimating reward associated with each action rather than the control law itself (which merely consists in choosing the action with the higher reward estimate, potentially with some added randomness [72]). Similarly, in go/no-go paradigms, the focus is on all-or-nothing inhibition of a motor response. A control theory framework such as PID control emphasizes the need to appropriately scale a continuous control action in relation to a reference error. Examining neural processing of reference errors (and potential dysfunction in this process) may offer new computational insights into mental health problems. Since reference errors inherently incorporate a motivational component (by comparing the current state to a goal), this approach will enable investigation of the integration of motivation into action and impairment of this process in mental health disorders.

Second, a control theory framework underscores the importance of system dynamics in estimating the current state of a system based on a model. While the Kalman filter and similar algorithms—including the RW algorithm, which can be seen as a streamlined version of the Kalman filter—have been applied to model associative conditioning, these applications often operate under the assumption that system variations arise from random fluctuations without any predictable pattern. Specifically, in the RW model, the next estimate is derived by blending the current estimate with the new measurement, effectively taking a weighted average. This means that RW relies on the immediate past rather than anticipating future changes based on underlying system dynamics. In contrast, the Kalman filter, before incorporating the new measurement, first makes a prediction of the next state based on a forward model, which can include the laws of motion or other predefined patterns of change. This allows the Kalman filter to anticipate changes rather than merely react to them, leading to a more comprehensive and reliable prediction mechanism. Research on motor control has provided evidence that this type of forward model-based prediction is a critical component of sensorimotor processing and is supported by specialized circuitry such as the cerebellum [68]. Furthermore, there is evidence of altered cerebellar activity in psychiatric disorders [73]. Investigating forward model-based prediction could therefore illuminate deficits in computation in mental health disorders which are not apparent from traditional reinforcement learning paradigms.

Third, binary-choice paradigms may make it difficult to disambiguate rapid, automatic processing from slow, deliberative processing. While dual system theories broadly differentiating these two types of processing are likely simplifications [74], careful modeling studies have demonstrated that computational tradeoffs are managed in the brain via complementary contributions from specialized network architectures in the cortex, hippocampus, and striatum [75]. A complex task such as a two-armed bandit may therefore reflect both a fast reinforcement learning contribution from the striatum as well as a slow working memory contribution from cortex, and measurement of striatal processing may be masked by the working memory overlay [76]. Continuous sensorimotor paradigms may help to precisely characterize deficits in rapid neural processing in mental health disorders because reactions to stimuli can be measured in real-time.

Finally, continuous sensorimotor paradigms enable collection of a large amount of data in a short time (i.e. 60 data points per second rather than a single data point every several seconds [77]). This can help improve the generally low reliability values for a wide range of behavioral tasks related to control and self-regulation [78], which is an important consideration for computational psychiatry (where reliable measures will be crucial to support clinical decision making [11]). The improved reliability achieved with continuous paradigms has already been demonstrated in psychophysics, in which control theory models of continuous tracking tasks have enabled calculation of perceptual parameters in much less time compared to traditional binary-choice paradigms [79, 80]. Taken together, control theory approaches may provide a better framework for real-world decision-making by focusing on continuous action spaces and system dynamics, allowing for a more nuanced understanding of mental health disorders. Moreover, this framework enables the collection of more granular, real-time data, improving the reliability of behavioral tasks and thereby supporting more robust clinical decision-making.

### PID control parameters as reliable markers of affect-related motor function

Given that PID control was initially developed based on observations of human ship pilots [29], it is unsurprising that PID control has subsequently been used successfully to model human performance in a variety of domains, including balance keeping [81], gait [82], and forearm control [83]. More recently, PID control models have been applied to measure individual differences in sensorimotor control and their relationship to subjective affect and to neural structure and function. In order to facilitate this, the rapid assessment of motor processing (RAMP) paradigm has been introduced [17, 77, 84]. In this task, participants perform a simulated one-dimensional driving task in which they drive a virtual car as quickly as possible and stop as close as possible to a stop sign without crossing the stop-line. The car is controlled according to a linear dynamical system, in which car velocity is proportional to joystick displacement at each time point. There are two versions of the RAMP paradigm, a joystick version [77] and a mobile version in which the car is controlled with a thumb on a device touchscreen [17]. In both versions, car position and velocity are recorded with a sampling window of 1/60 s, enabling reliable assessment of real-time, continuous motor control processing. Both tasks can be analyzed using a PD control model (the integral term of PID is not relevant as there is no external disturbance in the RAMP task). The model yields estimated *K*_*p*_ and *K*_*d*_ parameters for each trial. Given that participants control the position of a car rather than the position of a limb, the RAMP task should be understood as a task probing real-time goal-directed control rather than simple motor control.

As part of a larger study, 317 individuals comprised of 58 healthy controls and 259 individuals with mood and anxiety complaints completed the joystick RAMP task along with a battery of other assessments including self-report scales and neuroimaging [77]. *K*_*p*_ and *K*_*d*_ parameters were computed with very high reliability (split-half reliability of r = 0.98 for *K*_*p*_, r = 0.95 for *K*_*d*_, and r = 0.89 for residual *K*_*d*_, controlling for *K*_*p*_). These values are striking given the generally low reliability values for a wide range of behavioral tasks related to control and self-regulation [78]. Both *K*_*p*_ and *K*_*d*_ parameters were negatively associated with self-reported fear (measured using the Positive and Negative Affect Schedule-Expanded Form (PANAS X) [85]). These effects were hypothesized based on evidence for an effect of arousal on sensorimotor function as well as the relationships between fear, behavioral inhibition [86], and exaggerated error processing [87]. Supplemental analyses confirmed the specificity of the relationship between model parameters and fear (as opposed to other affects) [77]. Both *K*_*p*_ and *K*_*d*_ parameters were positively associated with volume in the dACC.

As part of the same larger study, 492 individuals comprised of healthy controls as well as individuals with mood and anxiety complaints, substance use disorders, and eating disorders completed the joystick RAMP task as well as a stop signal task in a functional magnetic resonance imaging (fMRI) scanner [84]. Individuals with higher *K*_*d*_, controlling for *K*_*p*_, on the RAMP task exhibited relatively less strategic adjustment after a stop trial on the stop signal task. Individuals with higher *K*_*d*_, controlling for *K*_*p*_, also exhibited increased activity in frontal and parietal regions as well as insula and caudate during response inhibition on the stop signal task.

In a separate study, 89 individuals completed the mobile RAMP task, of whom 66 completed the task a second time in a repeat session [17]. Both *K*_*p*_ and *K*_*d*_ exhibited high test–retest reliabilities (ICC3 0.81 and 0.78, respectively [17]). Additionally, both *K*_*p*_ and *K*_*d*_ were negatively associated with self-reported fear, replicating the finding from the joystick RAMP task.

The RAMP paradigm, analyzed using a PD control model, allows for a nuanced assessment of individual differences in continuous motor control processing. The high reliability of the *K*_*p*_ and *K*_*d*_ parameters indicates that this method could provide a robust measure of an individual's control mechanisms and potentially reveal unique patterns of sensorimotor control. Tasks such as the RAMP paradigm may enable a more nuanced view of computational deficits related to affect by distinguishing subcomponents of control processing related to the *K*_*p*_ and *K*_*d*_ parameters. *K*_*p*_ is a *driving* parameter that controls movement toward the goal based on current error. *K*_*d*_ is a *damping* parameter that prevents overcorrection by anticipating future error, avoiding oscillations around the goal state. Higher *K*_*d*_ (i.e. damping) is necessary in the setting of higher *K*_*p*_ (i.e. drive), with the combination helping to mitigate the inherent tradeoff between fast goal pursuit and overshoot. PD control therefore distinguishes an anticipatory component to error processing (*K*_*d*_) from the processing of current error (*K*_*p*_), where an appropriate balance between these components results in optimal control performance. The RAMP results suggest that subjective fear is related to exaggerated processing of anticipated error but reduced processing of current error [77], potentially building on previous results implicating altered error processing in anxiety [87]. A key distinction between drive and damping is that drive relies on an estimate of the current position, while damping relies on an estimate of the current velocity. Because velocity changes more quickly than position, damping may represent a more fundamental challenge and capacity limitation, such that damping capacity determines what level of drive is possible without overshoot [84]. Future research can help further disentangle subcomponents of control processing and their relationship with neural circuits and subjective affect.

The negative correlation between both *K*_*p*_ and *K*_*d*_ parameters and self-reported fear suggests a link between control processes and emotional states. This implies that our control mechanisms may be influenced by our emotional state or vice versa. It raises the possibility that interventions aimed at regulating emotional states could potentially impact sensorimotor control, and conversely, training to improve control mechanisms may have an effect on emotional states. The associations between *K*_*p*_ and *K*_*d*_ parameters and brain volume in the dorsal anterior cingulate cortex (dACC), as well as increased activity in frontal, parietal, insula, and caudate regions during response inhibition tasks, suggest that variations in these control parameters may be reflected in structural and functional differences in specific brain regions. This could have implications for understanding psychiatric disorders that affect these regions or are characterized by changes in volume or activity in these areas. The strong reliability of *K*_*p*_ and *K*_*d*_ parameters, along with their correlation with self-reported fear and specific brain characteristics, suggests that the RAMP paradigm could potentially serve as a useful diagnostic tool. For instance, it could help identify individuals at risk of anxiety disorders or other conditions associated with alterations in affect and stress. Furthermore, it could aid in tracking the effectiveness of interventions designed to improve these conditions.

While the RAMP paradigm has shown promise in estimating reliable parameters potentially relevant to stress- and anxiety-related disorders, it will also be important to develop other experimental paradigms using a control theory framework to improve understanding of control dysfunction in mental health disorders. In particular, future paradigms can further disentangle the separate contributions of state estimation and the control law, further investigate the effect of reward and punishment on control processing, or include arousal manipulations to examine the effect of affective state on control processing within individuals. Additionally, since subjective affect is only one component of stress- and anxiety-related disorders, it will be important to determine whether control dysfunction represents a transdiagnostic dimension which may be relevant across individual disorders (such as panic disorder, generalized anxiety disorder, and posttraumatic stress disorder).

### Control deficits in stress- and anxiety-related disorders

The empirical findings linking motor control processing to both self-reported fearful affect and to structure and function in brain regions relevant to anxiety disorders suggest there may be fundamental connections between control processes and the pathophysiology of these disorders. While a fuller understanding of these relationships will require further research, existing evidence about the neural underpinnings of control and the effect of arousal on control suggest a speculative framework for an account of control dysfunction in anxiety. In particular, dysfunction in control processes in the context of arousal modulation may play a crucial role in the development and maintenance of anxiety disorders. Specifically, excessive arousal may impair control performance on complex tasks, contributing to impairment associated with anxiety disorders. Given the possibility that similar control algorithms may govern parallel circuits processing not only motor control but also higher cognitive functions such as working memory, cognitive control, and emotion regulation, impairment in these functions may be caused by dysregulated control processes in anxiety disorders. Dysfunction of an underlying control circuit comprised of frontal and parietal cortex, basal ganglia, and cerebellum, modulated by the salience network (including insula, dACC and amygdala) and NE, could have global cognitive and affective consequences but could be reliably probed with continuous sensorimotor paradigms (given the much higher time-density of data with these paradigms compared to traditional behavioral tasks). The empirical and theoretical evidence suggests that dysregulated control processes, influenced by excessive arousal and involving a complex neural circuit, may be a fundamental mechanism underlying the cognitive and affective impairments observed in anxiety disorders. However, this hypothesis will need to be rigorously tested using continuous sensorimotor paradigms, which offer higher time-density data compared to traditional behavioral tasks.

Furthermore, control deficits may not only represent consequences of dysregulated arousal but play a causal role in anxiety disorders by disrupting emotion regulation, assessment of threat, and other cognitive and affective processes. For example, deficits in emotion regulation may lead to maladaptive coping strategies, increased susceptibility to stress, and a propensity for experiencing heightened levels of anxiety [88]. These deficits may arise from disruptions in parallel circuits that process motor control alongside working memory, cognitive control, and emotion regulation, possibly due to shared computational algorithms, which can be explored in future research.

**Fig. 1:**
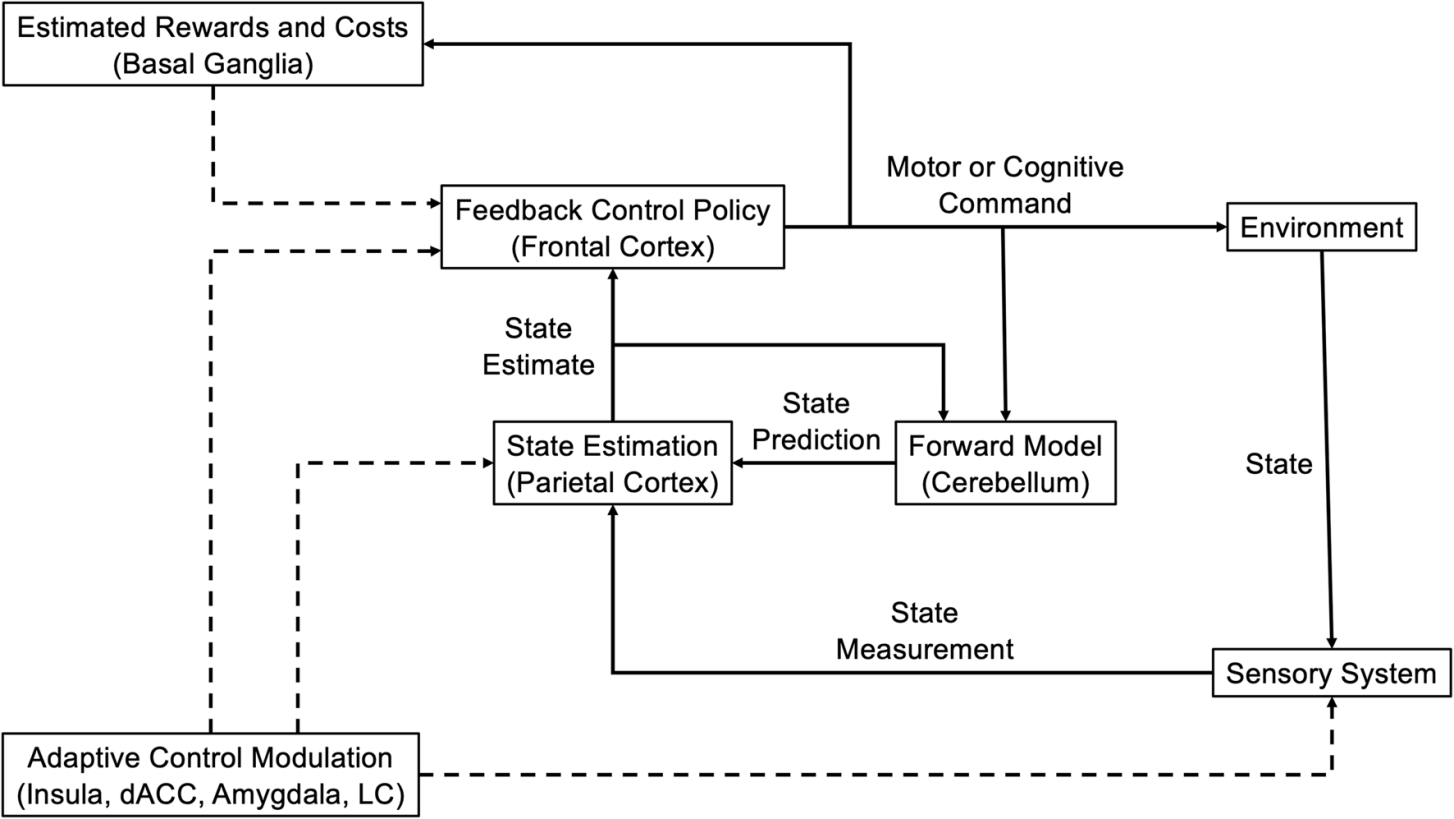
Neural Control Circuit. Frontal cortex implements a feedback control policy by comparing a reference or goal state to a current estimate of the state of the environment. This state estimate is produced in parietal cortex by combining noisy sensory measurements with state predictions generated by the cerebellum. This basic circuit is modulated by estimated reward and costs generated in basal ganglia and by arousal coordinated by the salience network [insula, dorsal anterior cingulate cortex (dACC), and amygdala] and locus coeruleus (LC). Dysregulated arousal in stress- and anxiety-related disorders can disrupt neural control processing, while altered control processing can cause dysregulation of the arousal response. Solid arrows illustrate the basic control circuit, while dashed arrows indicate modulation. Figure adapted with additions from Shadmehr and Krakauer [47]

The specific mechanism of control dysfunction in stress- and anxiety-related disorders may relate to the effect of arousal, via NE and potentially other neuromodulators, on state estimation. As discussed above, NE may increase the Kalman gain or a related parameter in neural state estimation, increasing the influence of new information. This may allow for faster responses in simple tasks, but reduce stability of state representations in complex tasks. Unstable internal state representations may prompt a compensatory reduction *K*_*p*_ and *K*_*d*_ parameters on complex tasks, sacrificing speed to maintain stability of motor output. Future computational studies can further clarify the underlying causes of control deficits related to anxiety, potentially yielding mechanistic insights that may be useful in developing new interventions. See Table 1 for a list of hypotheses emerging from control theory relevant to stress- and anxiety-related disorders. Future computational and empirical research will be needed to further formulate and test these hypotheses. See Fig. 1 for an illustration of a putative control circuit that may be disrupted in stress- and anxiety-related disorders.

**Table 1:** Control theory hypotheses relevant to stress- and anxiety-related disorders

Area of focus	Hypothesis description
Sensorimotor control and arousal	Sensorimotor control is modulated by arousal levels that affect the weighting of new sensory information in state estimation, leading to an inverted-U relationship with performance
Arousal in psychiatric disorders	Heightened arousal in psychiatric disorders, such as anxiety, disrupts environmental state estimates and control performance, measurable through sensorimotor paradigms
Domain-general control processes	Control theory models may explain neural operations in both sensorimotor and cognitive domains, with state estimates being central to driving adaptive actions
Arousal and abstract planning	Increased arousal levels potentially destabilize abstract state variables, impairing cognitive processes and planning in stress-related disorders
Control and arousal dynamics	Dysregulated arousal and control deficits are bidirectionally linked, influencing the progression and manifestation of stress and anxiety disorders

The hypotheses specified here will require future computational and empirical studies for precise formulation and testing

### Future directions

The robust findings from the RAMP paradigm and the empirical evidence linking control processes to stress- and anxiety-related disorders offer a compelling foundation for future research. Several avenues appear particularly promising. First, extending the RAMP paradigm to other psychiatric conditions beyond anxiety and mood disorders could provide a more comprehensive understanding of control deficits in a broader range of mental health issues. Second, longitudinal studies could elucidate whether changes in *K*_*p*_ and *K*_*d*_ parameters precede the onset of psychiatric symptoms, thereby serving as predictive biomarkers. Third, the role of neuromodulators like NE in state estimation and control processes warrants further investigation, especially in the context of arousal and emotional states. This could lead to pharmacological interventions targeting specific neuromodulatory pathways to ameliorate control deficits. Lastly, the development of computational models that integrate both sensorimotor control and higher cognitive functions could offer a unified framework for understanding the complex interplay between control processes and psychiatric disorders. These future directions have the potential to significantly advance the field of computational psychiatry, offering both mechanistic insights and practical tools for diagnosis and treatment.

## Conclusions

Control theory, with its roots in the early twentieth century, has proven to be an indispensable framework in biological sciences, and its significance is expected to grow exponentially with the evolution of computational neuroscience. The application of control theory to sensorimotor control processing has been notably successful, with emerging evidence suggesting that similar neural algorithms are likely to regulate cognitive and affective processes as well. Stress and arousal can be interpreted through the lens of adaptive control processes. In response to changing circumstances, these processes dynamically adjust the parameters on control algorithms to maintain optimal functioning. However, in stress- and anxiety-related disorders, this mechanism may malfunction, leading to impaired control performance and unstable internal state estimates, thereby hindering effective responses to environmental stimuli. In a reciprocal manner, disruptions in control processes that regulate cognition and emotion can contribute to hyper-reactivity to potential threats, resulting in an overactive stress and arousal response. This bidirectional relationship suggests a vicious cycle: impaired control processing escalates stress and arousal, which in turn further impairs control processing. Stress- and anxiety-related disorders can thus be conceptualized as disorders of control, trapped in a self-sustaining loop of dysfunction. Understanding and accurately measuring the computational underpinnings of these control processes could yield significant breakthroughs in managing these disorders.

Innovative paradigms such as the Rapid Assessment of Motor Processing (RAMP) task, which employs control theory principles, are already proving invaluable in providing reliable measures of individual control mechanisms and their relationship to affective states. Harnessing such approaches could lead to interventions designed to recalibrate these control systems, potentially breaking the vicious cycle of dysfunction. Ultimately, leveraging control theory to dissect and understand these disorders at a computational level could lead to the development of more targeted and effective interventions. These advancements hold significant promise for improving the lives of those afflicted with stress- and anxiety-related disorders, further highlighting the pivotal role of control theory in advancing our understanding and treatment of complex biological systems.

## Data Availability

Not applicable.
